# Current and Prospective Methods for Plant Disease Detection

**DOI:** 10.3390/bios5030537

**Published:** 2015-08-06

**Authors:** Yi Fang, Ramaraja P. Ramasamy

**Affiliations:** Nano Electrochemistry Laboratory, College of Engineering, University of Georgia, Athens, GA 30602, USA; E-Mail: fangyi@uga.edu

**Keywords:** food loss, plant pathogen, volatile organic compounds, sensor, enzyme, antibody, DNA/RNA, bacteriophage

## Abstract

Food losses due to crop infections from pathogens such as bacteria, viruses and fungi are persistent issues in agriculture for centuries across the globe. In order to minimize the disease induced damage in crops during growth, harvest and postharvest processing, as well as to maximize productivity and ensure agricultural sustainability, advanced disease detection and prevention in crops are imperative. This paper reviews the direct and indirect disease identification methods currently used in agriculture. Laboratory-based techniques such as polymerase chain reaction (PCR), immunofluorescence (IF), fluorescence *in-situ* hybridization (FISH), enzyme-linked immunosorbent assay (ELISA), flow cytometry (FCM) and gas chromatography-mass spectrometry (GC-MS) are some of the direct detection methods. Indirect methods include thermography, fluorescence imaging and hyperspectral techniques. Finally, the review also provides a comprehensive overview of biosensors based on highly selective bio-recognition elements such as enzyme, antibody, DNA/RNA and bacteriophage as a new tool for the early identification of crop diseases.

## 1. Introduction

Global food security as determined by the balance of global food production and demand has become an important international issue in recent years [1,2,3]. In 2008, an increase in food prices brought about a global crisis that caused political and economic instability in some developing countries [4]. It was estimated that the demand for food will continue to increase for another 40 years due to the continuous increase in human population. The projections also indicate that an additional 70% of food production is required by 2050 to meet the needs [5]. Currently, over one billion people are suffering from different situations of malnutrition due to lack of food supply and approximately twice that population do not have access to sufficient nutrients or vitamins to meet their daily nutrition needs [6]. The situation can be attributed to the continuous decline in agricultural land area that causes a decrease in productivity. Although decrease in agricultural productivity can be attributed to a variety of reasons, damage caused by pests and pathogens plays a significant role in crop losses throughout the world. The losses in crop yield due to pathogen infections range between 20% and 40% [7]. On average, pathogen-induced losses of maize, barley, rice and soybean are estimated to be around 12%, groundnuts and potatoes are estimated to be around 24% and wheat and cotton are estimated to be around 50% and 80%, respectively [8]. Post-cultivation losses due to diseases and sub-standard quality are estimated to be 30%–40%. Overall, the economic losses due to infections are estimated at 40 billion dollars annually in the United States alone [9,10]. In order to minimize the disease induced damage in crops during growth, harvest and postharvest processing, as well as to maximize productivity and ensure agricultural sustainability, advanced disease detection and prevention in crops are highly important.

## 2. Current Methods for Crop Disease Detection 

Detection and identification of diseases in crops could be realized via both direct and indirect methods. Direct detection of diseases includes molecular and serological methods that could be used for high-throughput analysis when large numbers of samples need to be analyzed. In these methods, the disease causing pathogens such as bacteria, fungi and viruses are directly detected to provide accurate identification of the disease/pathogen. On the other hand, indirect methods identify the plant diseases through various parameters such as morphological change, temperature change, transpiration rate change and volatile organic compounds released by infected plants.

### 2.1. Direct Detection Methods

Table 1 compares the available direct detection methods for plant pathogens based on their limit of detection, advantages and limitations. Each of these methods is discussed in detail in the following paragraphs.

#### 2.1.1. Polymerase Chain Reaction

In the years of 1984 and 1993, two Nobel prizes were awarded to J.F. Kohler and C. Milstein, and K. Mullis for development of monoclonal antibodies and amplification of nucleic acid sequences, respectively, using the technology of polymerase chain reaction (PCR). Based on the fidelity of DNA hybridization and replication, PCR was initially used for highly specific detection of diseases caused by bacteria and viruses [11]. Now it has been widely used for the detection of plant pathogens as well. In addition to the basic PCR technology, advanced PCR methods such as reverse-transcription PCR (RT-PCR) has also been used for plant pathogen identification due to its high sensitivity [12]. Multiplex PCR was proposed to enable simultaneous detection of different DNA or RNA by running a single reaction [13,14,15,16,17]. Real-time PCR platforms have also been used for on-site, rapid diagnosis of plant diseases based on the bacterial, fungal and viral nucleic acids [18,19]. Although PCR technique can provide high sensitivity and specificity due to the fidelity of DNA amplification, it is also limited by lack of operational robustness [20]. PCR depends on the efficacy of DNA extraction and the performance is affected by inhibitors present in the sample assay, polymerase activity, PCR buffer and concentration of deoxynucleoside triphosphate [20]. In addition, application of PCR for pathogen detection requires designing a primer to initiate DNA replication, which could limit the practical applicability of this technique for field sampling of diseases [19].

**Table 1:** Comparison of the current methods for detecting plant diseases resulting from bacterial pathogens.

Techniques	Limit of Detection (CFU/mL) [12]	Advantages	Limitations
PCR	10^3^–10^4^	Mature and common technology, portable, easy to operate.	Effectiveness is subjected to DNA extraction, inhibitors, polymerase activity, concentration of PCR buffer and deoxynucleoside triphosphate.
FISH	10^3^	High sensitivity.	Autofluorescence, photobleaching.
ELISA	10^5^–10^6^	Low cost, visual color change can be used for detection.	Low sensitivity for bacteria.
IF	10^3^	High sensitivity, target distribution can be visualized.	Photobleaching.
FCM	10^4^	Simultaneous measurement of several parameters, rapid detection.	High cost, overwhelming unnecessary information.

PCR: polymerase chain reaction; FISH: fluorescence in-situ hybridization; ELISA: enzyme-linked immunosorbent assay; IF: immunofluorescence; FCM: flow cytometry; CFU: colony forming unit.

#### 2.1.2. Fluorescence *in-situ* Hybridization

Another type of molecular detection technique is fluorescence *in-situ* hybridization (FISH), which is applied for bacterial detection in combination with microscopy and hybridization of DNA probes and target gene from plant samples [21]. Due to the presence of pathogen-specific ribosomal RNA (rRNA) sequences in plants, recognizing this specific information by FISH can help detect the pathogen infections in plants. In addition to bacterial pathogens, FISH could also be used to detect fungi and viruses and other endosymbiotic bacteria that infect the plant [22,23]. The high affinity and specificity of DNA probes provide high single-cell sensitivity in FISH, because the probe will bind to each of the ribosomes in the sample. However, the practical limit of detection lies in the range of around 10^3^ CFU/mL. In addition to the detection of culturable microorganisms that cause the plant diseases, FISH could also be used to detect yet-to-be cultured (so called unculturable) organisms in order to investigate complex microbial communities [24]. However, besides the advantages, FISH also has some pitfalls that compromise the technique’s potency for plant disease detection [24,25,26]. For example, false positive results with autofluorescence materials are a common problem that often lowers the specificity. Accuracy and reliability of FISH is highly dependent on the specificity of the nucleotide probes. Insufficient penetration, higher order structure of target or probe (e.g., three-dimensional rRNA, loop and hairpin formation and rRNA-protein interactions), low rRNA content, photobleaching could also cause false negative results and hence compromise the limit of detection [24,25,26].

#### 2.1.3. Enzyme-Linked Immunosorbent Assay

The enzyme-linked immunosorbent assay (ELISA) is another molecular method for identification of diseases based on antibodies and color change in the assay [27]. In this method, the target epitopes (antigens) from the viruses, bacteria and fungi are made to specifically bind with antibodies conjugated to an enzyme. The detection can be visualized based on color changes resulting from the interaction between the substrate and the immobilized enzyme. The performance of ELISA can be improved greatly with the application of specific monoclonal and recombinant antibodies which are commercially available [28,29]. Specific monoclonal antibodies have been used in ELISA to achieve lower limits of detection in the order of 10^5^–10^6^ CFU/mL [12]. For plant disease detection, tissue print-ELISA and lateral flow devices that enable detection have been fabricated for on-site detection. However, the sensitivity for bacteria is relatively low (10^5^–10^6^ CFU/mL, Table 1) making it useful only for the confirmation of plant diseases after visual symptoms appear but not for early detection before disease symptoms occur [12].

#### 2.1.4. Immunofluorescence

Immunofluorescence (IF) is a fluorescence microscopy-based optical technique used for the analyses of microbiological samples. The technique can also be utilized to detect pathogen infections in plant tissues. For this technique, plant samples are fixed to microscope slides in thin tissue sections. Detection is achieved by conjugating a fluorescent dye to the specific antibody to visualize the distribution of target molecule throughout the sample [30]. IF has been used to detect onion crop infection by a fungus *Botrytis cinerea* [31]. IF has also been combined with other techniques such as FISH for *Solanum dulcamara* detection which causes crown rot in potatoes [32]. Similar to FISH and other fluorescence-based techniques, a significant problem with IF is photobleaching which results in false negative results. However, the decrease of sensitivity due to photobleaching can be controlled by reducing the intensity and duration of light exposure, increasing the concentration of fluorophores, and employing more robust fluorophores that are less sensitive to photobleaching.

#### 2.1.5. Flow Cytometry

Flow cytometry (FCM) is a laser-based optical technique widely used for cell counting and sorting, biomarker detection and protein engineering. FCM is used for rapid identification of cells while cells pass through an electronic detection apparatus in a liquid stream. The advantage of this technology is the capability for simultaneous measurement of several parameters. The technique uses an incident laser beam and measures the scattering and fluorescence of the laser beam reflected from the sample. Although FCM has been primarily applied to study cell cycle kinetics and antibiotic susceptibility, to enumerate bacteria, to differentiate viable from non-viable bacteria, and to characterize bacterial DNA and fungal spores, it is still a relatively new technique for plant disease detection application [33]. FCM in combination with fluorescent probes has been applied for rapid detection of foodborne bacterial pathogens. Accurate detections within 30 min down to level of 10^4^ colony forming units (CFU) per milliliter have been reported [33]. FCM has been proven to be efficient for detection of soil borne bacteria such as *Bacillus subtilis* in mushroom composts [34]. In addition to bacterial detection, FCM has also been reported for viability evaluation as well [35].

### 2.2. Indirect Detection Methods

In addition to the direct methods discussed above, indirect methods based on plant stress profiling and plant volatile profiling have also been used for the identification of biotic and abiotic stresses as well as pathogenic diseases in crops. In this regard, new types of optical sensors that detect biotic and abiotic stresses in plants have been developed and reported in the literature [36,37,38,39]. The optical sensors provide detailed information based on different electromagnetic spectra and thus, enable prediction of the plant health [40]. Thermography, fluorescence imaging and hyperspectral techniques are among the most favorable indirect methods for plant disease detection [41].

#### 2.2.1. Thermography

Thermography allows imaging the differences in surface temperature of plant leaves and canopies. The emitted infrared radiation can be captured by thermographic cameras and color difference can be analyzed. Previous reports have demonstrated that the loss of water in plants regulated by stomata would be affected by phytopathogens [42,43,44,45,46]. The resulting disease can be monitored through thermographic imaging and the amount of water transpired can be determined, without the external temperature influences [44]. The temperature changes resulting from the plant pathogen infection have been reported by several research groups [42,43,44,45,46]. The thermographic detection of plant diseases can also be scaled up for direct detection of disease in plants [47]. Thermography is also a promising tool to monitor the heterogeneity in the infection of soilborne pathogens [48]. However, the practical applicability of thermography for disease monitoring is limited due to its high sensitivity to the change of environmental conditions during measurements. Additionally, thermographic detection lacks the specificity towards diseases, and therefore cannot be used to identify the type of infection or distinguish between diseases that produce similar thermographic patterns.

#### 2.2.2. Fluorescence Imaging

In this technique, the chlorophyll fluorescence is measured on the leaves as a function of the incident light and the change in fluorescence parameters can be used to analyze pathogen infections, based on changes in the photosynthetic apparatus and photosynthetic electron transport reactions [49,50]. Using this technique, temporal and spatial variations of chlorophyll fluorescence were analyzed for precise detection of leaf rust and powdery mildew infections in wheat leaves at 470 nm [50]. Although fluorescence measurement provides sensitive detection of abnormalities in photosynthesis, the practical application of this technique in a field setting is limited [51,52,53].

#### 2.2.3. Hyperspectral Techniques

Hyperspectral imaging can be used to obtain useful information about the plant health over a wide range of spectrum between 350 and 2500 nm. Hyperspectral imaging is increasingly being used for plant phenotyping and crop disease identification in large scale agriculture. The technique is highly robust and it provides a rapid analysis of the imaging data. Furthermore, hyperspectral imaging cameras facilitate the data collection in three dimension, with X- and Y- axes for spatial and Z- for spectral, which contributes to more detailed and accurate information about plant health across a large geographic area [40]. Hyperspectral techniques have been widely used for plant disease detection by measuring the changes in reflectance resulting from the biophysical and biochemical characteristic changes upon infection. *Magnaporthe grisea* infection of rice, *Phytophthora infestans* infection of tomato and *Venturia inaequalis* infection of apple trees have been identified and reported using hyperspectral imaging techniques [54,55,56].

#### 2.2.4. Gas Chromatography

A completely different non-optical indirect method for plant disease detection involves the profiling of the volatile chemical signature of the infected plants. The pathogen infections of plants could result in the release of specific volatile organic compounds (VOCs) that are highly indicative of the type of stress experienced by plants [57]. An infection by *Phytophthora cactorum*, the fungus that causes crown rot diseases in strawberries, results in the release of *p*-ethylguaiacol and *p*-ethylphenol as characteristic VOCs from the infected portion of the strawberry plant/fruit [57,58]. VOCs are also produced when green leaf plants are damaged pathogenically and mechanically. For example, green leaf volatiles (GLVs) such as *cis*-3-hexenol, *cis*-hexenyl acetate and hexyl acetate are reported to have been produced during mechanical damage to plant leaves, *i.e.*, during herbivore invasion [59]. Profiling of such VOC could be used as a means to identify the type and nature of infection and accordingly be used for disease diagnostics and confirmation [57]. The volatile signature of plants could be analyzed using gas-chromatography (GC) technique to analyze the presence of the specific VOC that is indicative of a particular disease [60]. To enhance the performance of compound separation and analysis, the gas chromatography is often combined with mass spectrometry (GC-MS) to identify unknown compounds in the volatile sample [61,62,63]. In comparison to the optical imaging-based detection methods discussed above, GC/GC-MS can provide more accurate information about the plant disease due to its high specificity. It also allows the detection of diseases at different stages based on the quantitative information collected from the VOC sample. However, unlike the imaging system which can directly obtain the data on-field, GC/GC-MS requires sampling of pre-collected VOC for a longer time before data analysis, which severely limits its on-field application.

Although various methods for plant disease identification have already been developed and some have been implemented, their application is limited due to multiple reasons: they are either time consuming, destructive, demand a skilled technician, require laboratory set-up, do not provide real-time monitoring (e.g., FISH, ELISA, IF, FCM, GC-MS) and/or display low specificity (e.g., imaging techniques). Growers are interested in a solution that could help them identify pathogen infections in crops in a rapid, real-time and non-destructive fashion so that timely intervention and preventative treatments can be performed to contain the infection and minimize the crop losses. This would allow the growers to save millions of dollars in fungicide costs, by allowing them to localize sprayings and timely applications rather than preemptive spray massive regions of crop field.

## 3. Detection of Plant Diseases Using Portable Sensors

A wide variety of sensors have been developed and commercialized for various applications including environmental monitoring and medical diagnostics. Depending on the operating principle of the sensor, the analytes could be detected using a sensor based on electrical, chemical, electrochemical, optical, magnetic or vibrational signals. The limit of detection could be enhanced by the use of nanomaterial matrices as transducers and the specificity could be enhanced by the use of bio-recognition elements such as DNA, antibody, enzymes *etc*.

### 3.1. Biosensor Platforms Based on Nanomaterials

Recent breakthroughs in nanotechnology enable the preparation of various nanoparticles and nanostructures with few technical hurdles. Nanoparticles display fascinating electronic and optical properties and can be synthesized using different types of materials for electronics and sensing applications [64]. For biosensing application, the limit of detection and the overall performance of a biosensor can be greatly improved by using nanomaterials for their construction. The popularity of nanomaterials for sensor development could be attributed to the friendly platform it provides for the assembly of bio-recognition element, the high surface area, high electronic conductivity and plasmonic properties of nanomaterials that enhance the limit of detection. Various types of nanostructures have been evaluated as platforms for the immobilization of a bio-recognition element to construct a biosensor. The immobilization of the biorecognition element, such as DNA, antibody and enzyme, can be achieved using various approaches including biomolecule adsorption, covalent attachment, encapsulation or a sophisticated combination of these methods. The nanomaterials used for biosensor construction include metal and metal oxide nanoparticles, quantum dots, carbon nanomaterials such as carbon nanotubes and graphene as well as polymeric nanomaterials. Nanoparticles have been utilized with other biological materials such as antibody for detecting *Xanthomonas axonopodis* that causes bacterial spot disease [65]. Gold nanoparticle-based optical immunosensors have been developed for detection of karnal bunt disease in wheat using surface plasmon resonance (SPR) [66]. In addition to single probe sensors, nano-chips made of microarrays which contain fluorescent oligo probes were also reported for detecting single nucleotide change in the bacteria and viruses with high sensitivity and specificity based on DNA hybridization [67]. Fluorescent silica nanoparticles (FSNPs) combined with antibody as a biomarker have been studied as the probe, which successfully detected plant pathogens such as *Xanthomonas axonopodis* pv. *Vesicatoria* that cause bacterial spot diseases in *Solanaceae* plant [65].

Quantum dots (QD) have also been used for biosensor construction for disease detection [68]. Due to their unique and advantageous optical properties, they have been used for disease detection using fluorescence resonance energy transfer (FRET) mechanism [69], which describes energy transfer between two light-reactive molecules. Many QD-FRET-based sensors have been developed for phytodisease detection such as the witches’ broom disease of lime (WBDL) caused by *Candidatus* Phytoplasma aurantifolia (*Ca.* P. aurantifolia). The immunosensor constructed showed a high sensitivity and specificity of 100%, and a detection limit of 5 *ca.* P. aurantifolia/µL [70]. Additionally, QD-FRET was also reported to detect the disease vectors. For example, Rhizomania, which is the most destructive disease in sugar beet, is caused by *beet necrotic yellow vein virus* (BNYVV). *Polymyxa betae* (Keskin), the only known vector of BNYVV, for transmission of the virus to the plants was successfully reported to be detected by QD-FRET-based sensor [71].

**Figure 1:**
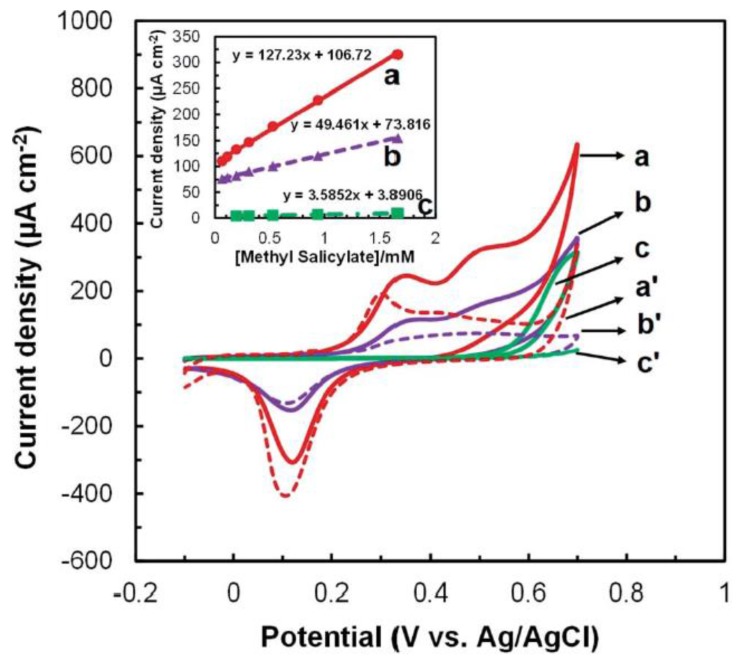
Cyclic voltammetry of (a) AuNP-Screen printed carbon electrode (SPCE), (b) planar gold and (c) SPCE in presence of 1.7 mM methyl salicylate. (a') AuNP-SPCE, (b') planar gold and (c') SPCE in the absence of methyl salicylate. The responses of current to methyl salicylate and sensitivity are shown in the inset. Figure is adopted from Ref. [77] with permission.

**Figure 2:**
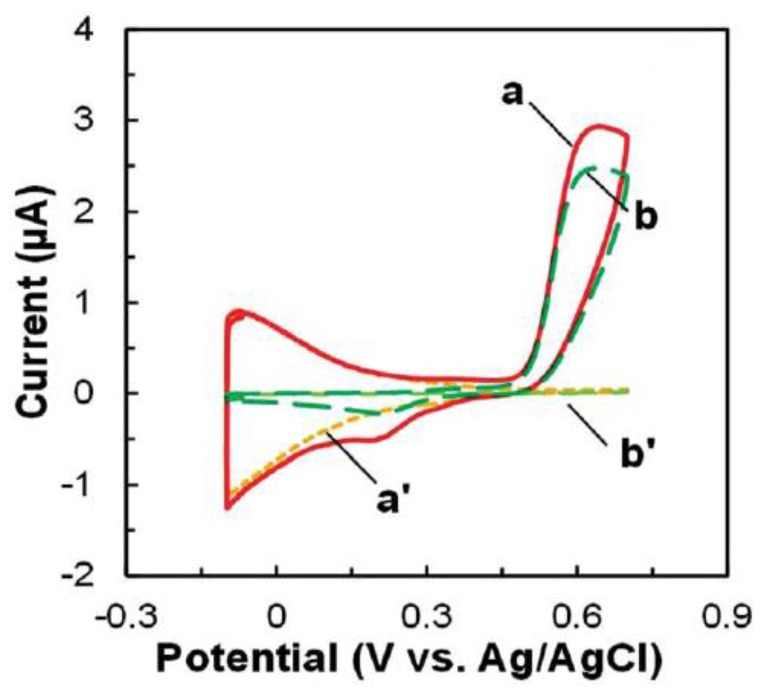
Cyclic voltammetry responses of (a and a') SnO_2_-Screen printed (SP) and (b and b') TiO_2_-SP (a and b) with and (a' and b') without the presence of 0.17 mM *p*-ethylguaiacol. Figure is adopted from Ref. [57] with permission.

In addition to QD-based sensors, the use of other novel materials for sensor fabrication have been explored in order to attain high sensitivity and low limits of detection [72,73,74]. Gold nanoparticles are widely used nanomaterials due to their high electroactivity and electronic conductivity for electron transfer [75,76]. Recently, nanomaterial-based electrochemical sensors have been reported for plant disease detection by our group [77]. The application of gold nanoparticle (AuNP) modified electrode has been reported by our group for the electrochemical detection of methyl salicylate, a key plant volatile organic compound released by plants during infections (Figure 1). [77]. Moreover, in addition to gold nanoparticles, semiconductive metal oxide nanoparticles have also been reported for VOC detection due to its advantages such as low cost, suitability for electron conduction for amperometric signal and the ease at which to obtain a desired size and shape. Our previous work has demonstrated the application of metal oxide nanoparticles such as SnO_2_ and TiO_2_ for VOC detection such as *p*-ethylguaiacol produced by infected strawberry (Figure 2) [57]. The limit of detection achieved was in the nanomolar concentration range [57]. In addition to the detection of plant VOC which is indicative of a particular disease, nanoparticles can also be used for detection of compounds released by the pathogen itself. Different types of phytopathogens—phytobacteria, viruses and fungi—have been detected through nanoparticle based amperometric biosensors [65,78,79].

### 3.2. Affinity Biosensors

Compared to the non-specific nanoparticle-based biosensors, inclusion of a bio-recognition element can greatly increase the specificity of the sensor. Consequently, other types of biosensors have been developed and among them affinity biosensors are popular. In affinity biosensors, the sensing is achieved based on the reaction of the bio-recognition element and the target analyte [80]. Affinity biosensors can be developed using antibody and DNA as recognition elements.

#### 3.2.1. Antibody-Based Biosensors

Antibodies are versatile and are suitable for diverse immunosensing applications. Antibody-based biosensor allows rapid and sensitive detection of a range of pathogens especially for foodborne diseases and this technique has already been developed for food safety monitoring. The antibody-based biosensors provide several advantages such as fast detection, improved sensitivity, real-time analysis and potential for quantification. Antibody-based biosensors hold great value for agricultural plant pathogen detection [81]. The biosensors enable the pathogen detection in air, water and seeds with different platforms for greenhouses, on-field and postharvest storages of processors and distributors of crops and fruits [81].

**Figure 3:**
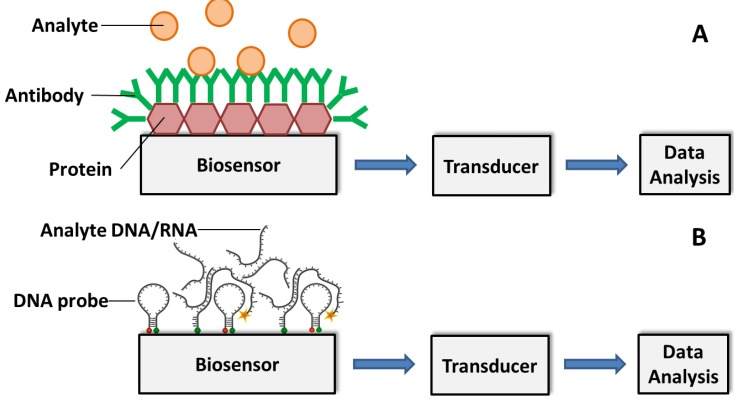
Schematic illustration of (**A**) antibody-based and (**B**) DNA/RNA-based biosensor for analyte detection. The specific combination of analyte and immobilized antibody (A) or DNA/RNA probe (B) produces a physicochemical change, such as mass, temperature, optical property or electrical potential. The change can be translated into a measurable signal for detection.

The principle of establishing antibody-based immunosensors lies in the coupling of specific antibody with a transducer, which converts the binding event (the specific binding of antibody modified on the biosensor with the antigen, e.g., pathogen of interest) to a signal that can be analyzed (Figure 3a). Most antibody-based biosensors use one of the following types of electrochemical transducers: amperometric, potentiometric, impedimetric and conductometric. Amperometric biosensing platforms use electric current signal resulting from the specific binding event [82,83]. Potentiometric biosensors on the other hand convert the biorecognition of an analyte into a voltage signal [82]. Impedimetric biosensors detect analyte by impedance change upon specific combination of the antibody and analyte. Based on the metabolic redox reactions of microorganisms, impedimetric biosensors are often used for biomass detection by microbial metabolism [84]. The conductometric biosensors are based on conductometric detection where the biological signal is converted to an electrical signal through a conductive polymer, such as polyacetylene, polypyrrole or polyaniline [80]. Other types of transducers (non-electrochemical) for affinity biosensing have been developed and reported including surface plasmon resonance (SPR), quartz crystal microbalance (QCM) and cantilever-based sensors. SPR-based sensors can measure the change in refractive index due to the attachment of the analyte to the metal surface (e.g., gold), which is usually modified with the recognition element of a conjugated ligand [85]. A QCM-based sensor detects a mass variation per unit area of the QCM crystal by measuring the change in frequency of a quartz crystal resonator. The QCM crystal is typically modified with a recognition element (e.g., antibodies) [86]. Similar to QCM-based sensors, cantilever-based sensors measure resonance frequency changes upon combination of the analytes and the sensor surface [81]. Based on the capability of detecting small analytes such as nucleic acid and proteins, cantilever-based sensors have been used for detection of pathogenic microorganisms [87].

During the past decade, many articles have been published demonstrating the capability of antibody-based biosensors for detection of plant pathogens such as *Cowpea mosaic virus*, *Tobacco mosaic virus*, *Lettuce mosaic virus*, *Fusarium culmorum*, *Puccinia striiformis*, *Phytophthora infestans*, orchid viruses and *Aspergillus niger* [86,88,89,90,91,92,93,94]. 11-mercaptoundecanoic acid was self-assembled on gold surface and then crosslinked with anti-maize chlorotic mottle virus (anti-MCMV) for MCMV disease detection. SPR response to MCMV solutions was evaluated over time with different concentrations from 1–1000 ppb. The limit of detection was evaluated to be approximately 1 ppb [85]. In recent years, antibody-based biosensor technology has seen tremendous progress upon implementation of nanotechnology based approaches for the sensor fabrication. Gold nanorods (AuNRs) functionalized by antibodies have been used to detect *Cymbidium mosaic virus* (CymMV) or *Odontoglossum ringspot virus* (ORSV) for rapid diagnosis of viral infections. The limits of detection (LODs) for CymMV and ORSV were reported to be 48 and 42 pg/mL, respectively, in leaf saps [95]. The detection of CymMV and ORSV was not only reported by SPR technique, but also by QCM technique. The QCM technique was able to detect each of the orchid viruses as low as 1 ng [86]. Other nanomaterials made of polymers such as polypyrrole (PPy) nanoribbon modified chemiresistive sensors were fabricated by a lithographically patterned nanowire electrode position (LPNE) technique. The fabricated biosensor was investigated for the detection of *Cucumber mosaic virus* (CMV) and displayed excellent sensitivity with a detection limit of 10 ng/mL [96]. The limits of detection of the current antibody based biosensors are proved to be approximately two orders of magnitude higher than that of conventional ELISA methods [97]. Apart from the common abiotic material for antibody-based biosensor fabrication, biosensors based on living cells are characterized by low limit of detection, high specificity and rapid response time. A novel portable cell biosensor system for detection of *Potato virus Y* (PVY), *Cucumber mosaic virus* (CMV) and *Tobacco rattle virus* (TRV) was fabricated by immobilizing the vero cells carrying virus specific antibodies on their membranes. This study demonstrated an important step towards the development of a portable plant virus detection system suitable for on-field application [98].

Although the mechanisms, advantages and applications of antibody-based biosensors for plant disease detection have been highlighted, it is important to discuss the limitations. Since many biosensors based on antibodies focus on specific binding with a particular antigen, issues such as the exposure of a bacterial strain to environmental stress (pH and temperature), could cause errors in the measurement. In addition, the immobilization of large bacteria and fungi, whose diameters exceed the SPR range, might compromise the detection. More importantly, antibodies are vulnerable and are easy to get denatured, which requires specific environment (pH, temperature, *etc.*) for storage, otherwise, the behavior of antibody-based sensor will also be compromised due to the deterioration of antibody over time [84].

#### 3.2.2. DNA/RNA-Based Affinity Biosensor

A recently developed new type of affinity biosensor uses nucleic acid fragments as elements for pathogen detection. The detection of specific DNA sequence is of significance in a variety of applications such as clinical human disease detection, environmental, horticulture and food analysis. Due to the possibility of detection at a molecular level, the DNA-based biosensor enables early detection of diseases before any visual symptoms appear. The application of specific DNA sequences has been widely used for detection of bacteria, fungi and genetically modified organisms. Based on the specific nucleic acid hybridization of the immobilized DNA probe on the sensor and the analyte DNA sequence, DNA-based biosensor allows rapid, simple and economical testing of genetic and infectious diseases. The most commonly adopted DNA probe is single stranded DNA (ssDNA) on electrodes with electroactive indicators to measure hybridization between probe DNA and the complementary DNA analyte [99]. There are four major types of DNA-based biosensors depending on their mode of transduction: optical, piezoelectric, strip type and electrochemical DNA biosensors. Optical DNA biosensors transduce the emission signal of a fluorescent label. The detection of DNA analyte is realized through a variation in physio-chemical properties such as mass, temperature, optical property and electrical property as a result of double-stranded DNA (dsDNA) hybridization occurs during the analyte recognition (Figure 3b). The optical DNA-based biosensors can be further classified to three subtypes—molecular beacons (MB), surface plasmon resonance (SPR) and quantum-dots. Unlike the optical type, piezoelectric DNA biosensors detect the analyte using a quartz crystal that oscillates at a specific frequency at an applied oscillating voltage. Detection of DNA hybridization can also be realized through nanoparticle-based colorimetric detection provided by strip type DNA biosensor. Electrochemical measurements are used for sequence-specific detection of analyte DNA by electrochemical DNA-based biosensors. The current change with constant applied potential can be monitored and used for interpretation of DNA hybridization in amperometric electrochemical DNA biosensor [99]. Bacterial pathogens are detectable by DNA-based biosensors due to their unique nucleic acid sequence, which can be specifically hybridized with the complementary DNA probe. The recognition of analyte DNA is dependent upon the formation of stable hydrogen bonds between the DNA probe and analyte DNA sequence. This is different from the antibody-based biosensors where hydrophobic, ionic and hydrogen bonds play a role in the stabilization of antigen-antibody complex.

**Figure 4:**
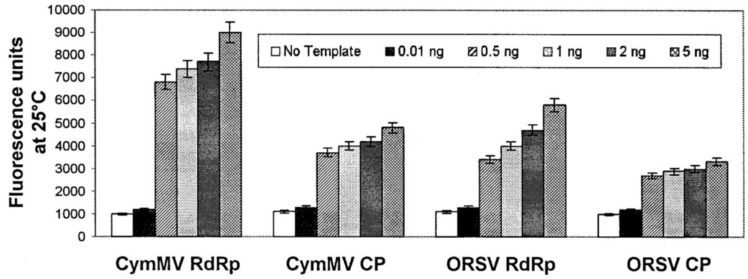
The fluorescence intensity of the four molecular beacons (CymMV RdRp, CymMV CP, ORSV RdRp and ORSV CP) with 0.01, 0.5, 1, 2, and 5 ng of purified viral RNA. The fluorescence intensity of all molecular beacons increased significantly and the limit of detection of purified viral RNA was estimated to be 0.5 ng. Figure has been adopted from Ref. [99].

**Figure 5:**
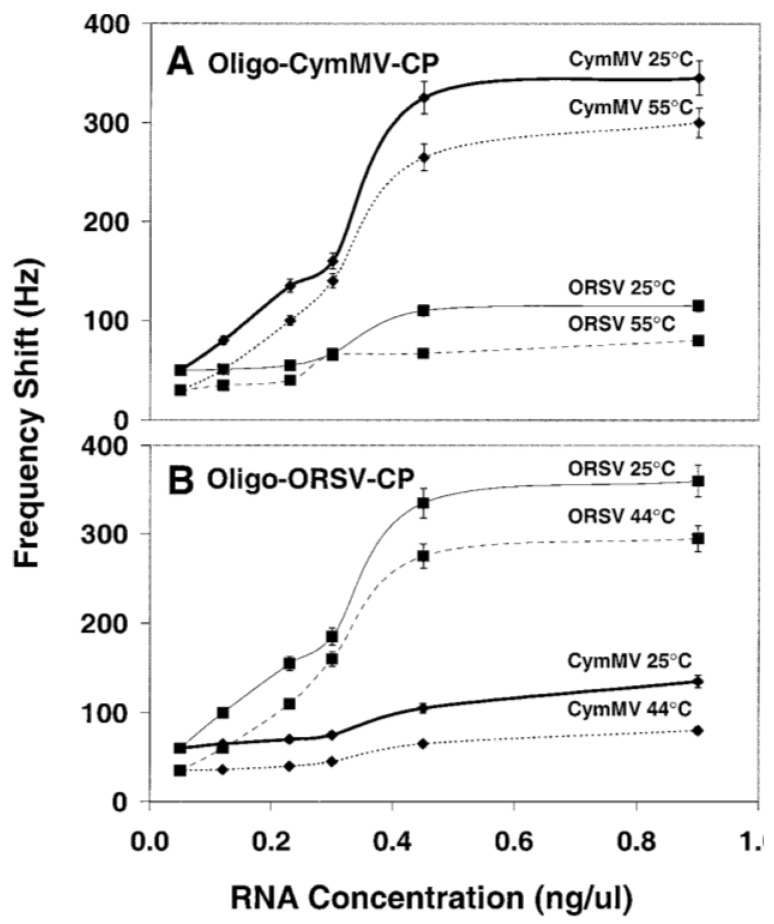
Sensitivity and specificity of (**A**) CymMV coat protein (CP) and (**B**) ORSV-CP QCM DNA biosensors upon incubation with increasing concentrations of viral RNA at different temperatures. Figure has been adopted from Ref. [100].

In addition to DNA-DNA hybridization for bacterial detection, the specific hybridization of DNA and complementary RNA was also exploited for the detection of plant viruses by molecular beacons and QCM techniques. Two orchid viruses—*Cymbidium mosaic virus* (CymMV) and *Odontoglossum ringspot virus* (ORSV)—have been detected with specific oligonucleotide probes with a fluorescent moiety attached to one end of DNA while a quenching moiety attached to the opposite end. Four such molecules have been designed and this technique has been successfully applied to detect viral RNA of both orchid viruses with limits of detection as low as 0.5 ng of viral RNA in 100 mg orchid leaves (Figure 4) [99]. CymMV and ORSV were also detected by QCM DNA-based biosensor with a designed DNA probe modified with a mercaptohexyl group. The limits of detection of CymMV and ORSV were as low as approximately 1 ng in purified RNA and 10 ng in the crude sap (Figure 5) [100]. Although the application of DNA-based biosensors for plant disease detection is promising, PCR may have to be performed prior to the probing process due to the small quantity of nucleic acid present in the bacteria cells [101]. The limitations of DNA based biosensors include the requirement for the synthesis of specific DNA probe, amplification of DNA, high cost (DNA-based molecular beacons) and unsuitability for real-time detection (DNA-based piezoelectric biosensor).

### 3.3. Enzymatic Electrochemical Biosensors

**Figure 6:**
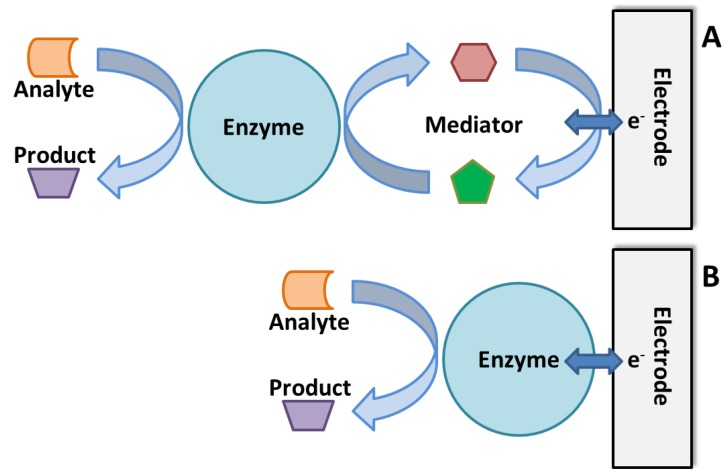
Schematic illustration of enzymatic biosensor based on (**A**) mediated electron transfer and (**B**) direct electron transfer (DET).

The use of enzyme as bio-recognition element can provide highly selective detection of the target analyte due to the high specificity of enzymes towards the analyte. An enzyme specific for the analyte of interest is immobilized on the nanomaterial modified-electrode. The amperometric detection is based on the bio-electrocatalytic reaction between the target analyte and electrode, which results in an electrical signal (current) that can be used for quantitative detection of the analyte. The amperometric signal can be obtained through either direct or mediated electron transfer based electrochemical reactions (Figure 6). Unlike other types of biosensors, which are not widely commercialized, the enzymatic electrochemical biosensors have been successfully commercialized, thanks to the invention of glucose biosensors, which are widely used in personal diabetes monitors [102]. A similar biosensing methodology can be adopted for plant pathogen detection, food quality detection and environmental monitoring [103]. For plant pathogen detection, enzymatic biosensors could be used if the target VOC could be collected in the form of a liquid sample. Previous studies have shown that several of phytohormones are catabolized by redox enzymes, offering prospects for using these enzymes for the development of highly selective enzyme-based biosensors for detecting plant chemicals [104]. Our previous work has already proved the detection of methyl salicylate with a bi-enzymatic system [105]. Many of the VOCs produced by infected crops are alcohols and aldehydes such as *cis*-3-hexen-1-ol and *trans*-2-hexanal, which can be catalyzed by alcohol dehydrogenase enzymes. Accordingly, these enzymes can be used for the development of biosensors for the detection of alcohol or aldehyde based VOCs which are specific to the infection. A summary of the different volatiles known to be released due to plant stresses are listed in Table 2 [60]. In addition to those specific volatile organic compounds, the common phytohormones such as auxin, cytokinins and gibberellins which are indicative of plant health could also be deactivated by oxidases. Gibberellin is deactivated by GA-2-oxidases which provides the potential for fabrication of gibberellin detection for plant disease prediction [104,106]. Although enzyme-based biosensors usually provide high sensitivity and specificity for the detection, stability of enzymes is of major concern. In addition, the enzyme catalysis varies with factors such as temperature and pH which compromises the accuracy of the biosensor.

**Table 2:** VOCs emitted from whole, intact tomato plants or detached leaves, and biotic stress causing agents responsible for increases in VOC emissions [60].

Volatiles	Biotic Stress Causing Agents that Increase VOC Emissions
*cis*-3-hexen-1-ol	*Botrytis cinerea*, *Spodoptera littoralis*, *Lirimyza huidobrensis*, *Spodoptera exigua*, *Manduca sexta*, *Macrosiphum euphorbiae*, *Helicoverpa armigera*
*trans*-2-hexanal	*Botrytis cinerea*, *Spodoptera littoralis*, *Lirimyza huidobrensis*, *Spodoptera exigua*, *Manduca sexta*, *Helicoverpa armigera*
Methyl salicylate	*Botrytis cinerea*, *Spodoptera littoralis*, *Tetranychus urticae*, *Manduca sexta*, *Macrosiphum euphoria*, *Tobacco mosaic virus*

### 3.4. Bacteriophage-Based Biosensors

Bacteriophage is a virus, composed of protein capsid that encapsulates a DNA or RNA genome. It infects the bacteria and replicates within the bacteria and finally lyses the bacterial host to propagate. Being able to lyse the bacteria, bacteriophage has been widely studied and used in phage therapy to cure bacterial infections [107]. Phage therapy has been used for not only human diseases, but also plant disease control. In addition to phage therapy, bacteriophage is also emerging as a promising alternative for pathogen detection due to its high sensitivity, selectivity, low cost and higher thermostability [108,109,110]. Upon the interaction between the bacteriophage and the target analyte, the impedance of charge transfer reactions at the interface changes which is used as a signal for detection.

**Figure 7:**
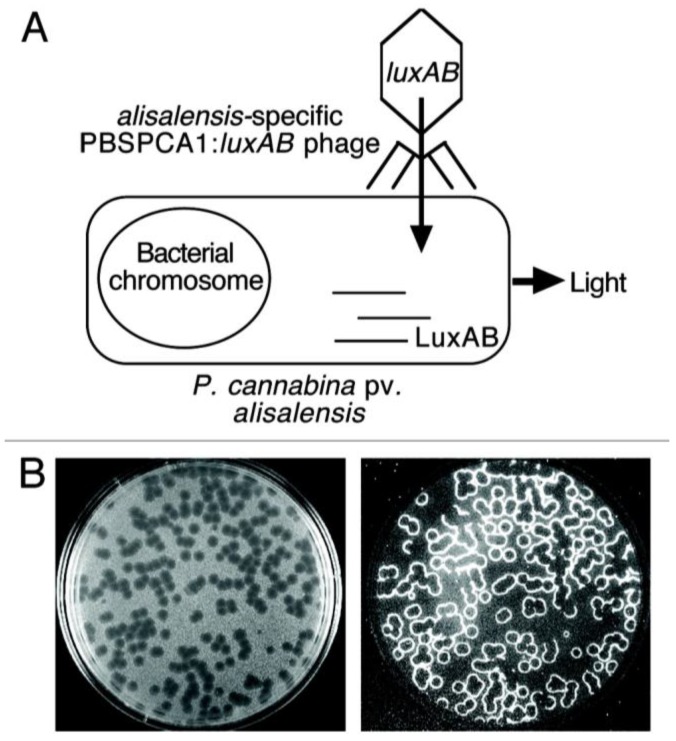
Schematic illustration of (**A**) *P. cannabina* pv. *alisalensis* detection and (**B**) bioluminescent plagues with examination under light (left) and dark field (right) illumination. The bioluminescence at the plague periphery (phage/cell interface) indicates phage-infected pathogens. Figure has been adopted from Ref. [114].

Bacteriophages have demonstrated to be successful in controlling plant pathogens recently such as *Dickeya solani*, the bacterial infecting of potatoes and tomatoes [111,112]. Tlili *et al.* reported the limit of detection for *E. coli* by using T4 phage and achieved 8 × 10^2^ CFU/mL in less than 15 min and 10^2^ CFU/mL within 40 min by impedimetric and loop-mediated isothermal amplification (LAMP) method respectively [113]. D. A. Schofield reported a phage-based diagnostic assay for detecting and identifying *Pseudomonas cannabina* pv. *Alisalensis* from cultures and diseased plant samples [114]. The bacterial *luxAB* reporter genes (encoding the luciferase) were integrated into *P. cannabina* pv *Alisalensis* phage PBSPCA1. In the presence of target pathogen cell, the *lux AB* can be expressed in the host cell due to specific infection through the phage. The expressed luciferase results in the light emission in presence of n-decanal, oxygen and flavin mononucleotide (Figure 7) [114]. In addition to *P. cannabina* pv. *alisalensi*, bacteriophages that specifically bind with other pathogens are also discovered and reported, such as *Pseudomonas syringae* pv. *Actinidiae*, which causes bacterial canker of kiwifruits, and *Ralstonia solanacearum*, a soilborne bacterium that is the causative agent of bacterial wilt in many important crops [115,116,117]. The progress in discovering more bacteriophages provides the possibility for fabrication of more bacteriophage-based sensors for plant disease detection.

The advantages of using bacteriophage as the recognition element for biosensors are its high selectivity and low cost of the phage. Furthermore, compared to the antibody-based sensor, bacteriophage-based sensors are more thermostable which allows the detection in different temperature ranges and longer shelf life. Bacteriophage-based biosensors are also capable of differentiating the live and dead bacterial pathogens which decreases the false positive signals during measurement. However, measuring the pathogen in a real sample using phage-based biosensors could take a longer time because of the complex sample preparation requirement [113]. Apart from that, bacteriophage-based sensor can only be fabricated for detection of bacteria rather than fungi and viruses which severely limits its application for the majority of crops that are affected by fungal pathogens.

## 4. Challenges and Future Directions

Besides the unique advantages offered by the various disease detection methods for plant disease detection application, each method has its own limitations. When it comes to direct disease detection methods, PCR has displayed its ability in detecting plant pathogen with high sensitivity, however, it requires designing specific primers to amplify DNA for detecting different pathogens. The cost prohibitive procedure thus limits its application only to laboratory settings and high value target analytes [19]. In addition, parameters like polymerase activity, buffer concentration of deoxynucleoside triphosphate can bring uncertainty to the result [20]. On the other hand, as sophisticated equipment in the laboratory-based detection techniques, portable PCR and its specialized types such as RT-PCR, real-time PCR have been used for on-field detection. We anticipate the application of PCR will be continued in the future. Although the application of ELISA for plant disease detection is not much reported, fabrication of test strips based on ELISA will be an innovative strategy for plant disease detection in the future due to its visible color change signal. However, the application may only be confined to virus detection due to the excellent sensitivity of ELISA for viruses (Table 3). The application will be compromised for bacterial infections due to its poor sensitivity, on the other hand (Table 1). Although FISH and IF provide excellent sensitivity (Table 3), they are laboratory-based techniques which require skilled personnel to operate. Additionally, complex sample preparations and professional data analysis are required. The same holds true for GC-MS despite its ability to provide quantitative determination of VOCs produced by infected plants. While offering accurate data for disease detection, FCM provides overwhelming and sometimes unnecessary data which complicates the data analysis and requires professional and experienced technicians for interpreting the results of detection. Further, the expensive instrumentation makes it less likely for on-field application.

Among the indirect imaging techniques for plant disease detection, the hyperspectral technique is the most promising due to its robustness, high specificity and rapid data analysis. Hyperspectral imaging could be obtained using a camera fitted to an unmanned aerial vehicle (UAV) for collecting data across a wider area of agricultural field. Thermography and fluorescence imaging, being non-specific and susceptible to ambient environment, therefore are less suitable for on-field crop disease detection [51,52,53]. Affinity sensors, although extensively studied, still remain in the laboratory in most cases. This can be explained by the sensor’s lack of the robustness, which is primarily due to the deterioration of antibody and DNA/RNA probe over time and the requirement for coupling with optical instruments. DNA-based molecular beacon biosensors could be potentially applied for on-field testing due to their properties such as real-time testing and low limit of detection (Table 3) given that the proper DNA probe is produced for this purpose. Also, the lower limit of detection provided by bacteriophage-based sensors is also promising, to be applied for live bacterial pathogen detection with the discovery of more bacteriophages [113]. However, the technique cannot be applied for fungal and viral pathogen detections. On the other hand, enzyme-based electrochemical biosensors constructed using nanomaterial platforms have higher specificity and applicability for real-time detection than other available technologies (Table 3). The electrochemical enzymatic biosensor also enables the fabrication of small-scale, portable and easy-to-use devices for detection. However, the current challenges reside in the identification, production and purification of enzymes specific to each target VOC produced by the infected plants. It is also promising that the enzymatic and affinity sensors can be fabricated for multiple pathogen detection, similar to electronic noses, rather than for single pathogen detection.

**Table 3:** Comparison of affinity biosensors, enzymatic biosensors, nanomaterial modified sensors and ELISA.

Techniques	Limit of Detection	Advantages	Disadvantages	Ref.
Nanomaterial sensor	35 nM (*p*-ethylguaiacol)	Real-time, easy to fabricate, stable	Low specificity compared to enzyme, DNA, antibody based biosensors	[57]
Enzymatic biosensor	0.98 µM (methyl salicylate)	High specificity, real-time	Interference from pH change	[105]
DNA-based piezoelectric biosensor	10 ng (virus)	Low cost	Not real-time, DNA may need to be amplified	[100]
DNA-based molecular beacon biosensor	0.5 ng (virus)	Real-time, low limit of detection	High cost, DNA may need to be amplified	[118]
Antibody-based quartz crystal biosensor	10 ng (virus)	Low cost	Not real-time	[86]
ELISA	2.5 ng (virus)	Low cost, low limit of detection for virus	Not real-time for bacteria, poor sensitivity for bacteria	[119]

## 5. Conclusions

In this article, we reviewed the currently existing methods for detection of plant diseases caused by pathogens such as bacteria, viruses and fungi. Although established methods such as PCR, FISH, ELISA, IF, FCM and GC-MS are already available and widely used for plant disease detection, they are relatively difficult to operate, require expert technicians and are time consuming for data analysis. In addition, most of these methods cannot provide real-time detection which makes them less suitable for on-field testing and early warning systems. On the other hand, imaging techniques such as thermography and fluorescence imaging, although they have been used on-field for disease detection, are proved to be susceptive to parameter change of the environment and lack of specificity of each type of disease. The untamed potential of various biosensors for plant disease detection has been comprehensively reviewed in this article. The advent of nanotechnology has resulted in the advancement of highly sensitive biosensors due to modern nanofabrication techniques. The specificity of the biosensors could be greatly enhanced by the use of enzymes, antibodies, DNA and bacteriophage as the specific recognition element.
